# Optimization of Extraction Process and Antioxidant and Antibacterial Activities of Total Phenolics from *Thalictrum baicalense* Turcz

**DOI:** 10.3390/pathogens15101088

**Published:** 2026-10-16

**Authors:** Lili Wu, Boyuan Liu, Jiawen Yao, Sha Li

**Affiliations:** School of Bioengineering, Zunyi Medical University, Zhuhai 519041, China; 13016300649@163.com (L.W.); 15175917615@163.com (B.L.); 18286081096@163.com (J.Y.)

**Keywords:** *T. baicalense*, response surface methodology, total phenolics, antioxidant activity, antibacterial activity

## Abstract

*Thalictrum baicalense* Turcz (*T. baicalense*) is a traditional medicinal plant. In this study, response surface methodology was used to optimize the ultrasound-assisted solvent extraction of total phenolics from *T. baicalense*, and its antioxidant activity, antibacterial activity, and potential antibacterial mechanism were evaluated. The optimal extraction conditions were determined as follows: ultrasonic power 120 W, solid-to-liquid ratio 1:10 (g:mL), extraction time 72 min, temperature 32 °C, and ethanol concentration 49%. Under these conditions, the total phenolic extraction yield reached 15.116 mg gallic acid equivalents per gram of plant powder (mg GAE/g). Antioxidant assays showed that the scavenging rates against 2,2-diphenyl-1-picrylhydrazyl (DPPH), 2,2′-azino-bis (3-ethylbenzothiazoline-6-sulfonic acid) (ABTS^+^), and hydroxyl radicals were 83.41%, 94.69%, and 78.95% at the total phenolic concentrations of 33.33, 20, and 3.85 μg GAE/mL, respectively, and were comparable to those of vitamin C. Antibacterial evaluation revealed that the total phenolic extract exhibited significant inhibitory activity against the Gram-positive bacterium *Staphylococcus aureus*, with an inhibition rate of 90.34% at a concentration of 570 μg GAE/mL, whereas weaker activity was observed against the Gram-negative bacterium *Escherichia coli*. Furthermore, crystal violet staining assay and scanning electron microscopy observation suggested that the extract reduced biofilm biomass and caused morphological alterations. In addition, treatment with the extract significantly decreased intracellular ATP levels in both strains, suggesting a possible effect on bacterial energy metabolism. However, these findings do not establish a specific antibiofilm mechanism or comparable efficacy to carbenicillin. Collectively, these findings provide a preliminary scientific basis for the potential application of natural bioactive components from *T. baicalense* in the fields of antioxidant and antibacterial agents.

## 1. Introduction

*Thalictrum baicalense* Turcz (*T. baicalense*) is a species of the genus *Thalictrum* in the family Ranunculaceae. This genus comprises approximately 200 species worldwide [1], of which 99 species and 22 varieties have been found in China, with 43 species used as medicinal plants in Chinese folk medicine [2,3]. The genus is widely distributed across Asia, Africa, Europe, South America, and North America [3]. In China, it is mainly found in Gansu, Sichuan, Yunnan, Tibet, Jilin, Henan, Qinghai, Ningxia, and Shaanxi [4]. Notably, the regions of Sichuan, Yunnan, Henan, and Gansu in China exhibit remarkable regional species diversity within this genus. Plants of this genus possess various pharmacological activities, including antibacterial, antioxidant, antiviral, and antitumor effects, and are commonly used in folk medicine for the treatment of hypertension and enteritis, as well as being employed as diuretics and stomachics [5,6,7,8]. Previous studies have demonstrated that other species of the same genus, such as *Thalictrum foliolosum* and *Thalictrum foetidum*, possess considerable antioxidant and antibacterial activities, which are closely related to their contents of total phenolics, flavonoids, alkaloids, and other bioactive constituents [9,10,11]. However, current research on *T. baicalense* has mainly focused on its alkaloids, fungal associations, and chloroplast genome [12,13], while the extraction methods for its total phenolic content and their antioxidant and antibacterial properties remain largely unexplored.

Plant-derived phenolic compounds, including flavonoids, flavonols, isoflavones, flavanones, chalcones, and flavan-3-ols, exhibit broad-spectrum antibacterial potential [14,15], and the escalating crisis of antibiotic resistance has made the discovery of novel antimicrobial agents from natural products a research priority [16,17,18]. *T. baicalense*, as a medicinal plant that has not yet been systematically studied, may harbor phenolic constituents of considerable developmental value. Therefore, screening for efficient and safe antibacterial active ingredients from this plant could not only provide new strategies for tackling antibiotic resistance but also lay the groundwork for the development of natural antibacterial alternatives. To achieve this goal, it is first necessary to establish an efficient extraction method for phenolics. Conventional extraction techniques, such as maceration, reflux extraction, Soxhlet extraction, and decoction [19,20] are time-consuming, require large volumes of solvents, and the prolonged heating involved tends to degrade thermosensitive components [21,22]. In contrast, ultrasound-assisted solvent extraction (UASE) employs cavitation effects to disrupt plant cell walls, thereby effectively shortening extraction time and enhancing extraction yield [23,24]. Meanwhile, response surface methodology (RSM), as a modern experimental design approach, enables the optimization of extraction parameters through the construction of mathematical models, significantly improving extraction efficiency while reducing experimental costs and time consumption [25].

Although RSM has been widely applied to optimize the extraction of phenolics from various plant materials, no study to date has reported its use for optimizing UASE conditions for the extraction of total phenolics from *T. baicalense*. Moreover, the antioxidant and antibacterial potential of the total phenolic constituents from this plant has not yet been systematically evaluated. To address this gap, the present study aims to employ RSM to optimize the UASE parameters, specifically ultrasonication time, ultrasonication temperature, and ethanol concentration, so as to enhance the extraction efficiency of total phenolics from *T. baicalense*. Additionally, this study represents the first systematic assessment of the antioxidant and antibacterial activities of the ethanolic extracts, along with a preliminary exploration of their antibacterial mechanism. The findings are expected to provide a theoretical foundation for the functional development and utilization of this medicinal plant.

## 2. Materials and Methods

### 2.1. Materials and Reagents

Whole herbs of *T. baicalense* were collected from Chengdu City, Sichuan Province, China. The voucher specimen (2022-dp-5) was identified by Zhang Jun from Kunming GenPHYTech Co., Ltd. (Kunming, China) and was deposited at Zunyi Medical University, China. Gallic acid (purity ≥ 98%, HPLC grade), anhydrous sodium carbonate, Folin-Ciocalteu reagent, vitamin C, ferrous sulfate, potassium persulfate, salicylic acid, 2,2-diphenyl-1-picrylhydrazyl (DPPH), and 2,2′-azino-bis (3-ethylbenzothiazoline-6-sulfonic acid) diammonium salt (ABTS^+^) were all obtained from Yuanye Biotechnology Co., Ltd. (Shanghai, China). Yeast extract powder, bacteriological peptone, and sodium chloride were all supplied by Huankai Microbiological Technology Co., Ltd. (Guangzhou, China). The ATP assay kit was obtained from Beyotime Biotechnology (Shanghai, China). Lysozyme was purchased from Sangon Biotech Co., Ltd. (Shanghai, China).

### 2.2. Bacterial Strains

The bacterial strains used in this study, *Escherichia coli* (*E. coli*) BW25113 and *Staphylococcus aureus* (*S. aureus*) ATCC 29213, were preserved in Luria-Bertani (LB) broth (containing 1% tryptone, 1% NaCl, and 0.5% yeast extract) supplemented with 17% (*v*/*v*) glycerol and stored at −80 °C until use.

### 2.3. UASE of Total Phenolic Extract from T. baicalense

Total phenolic compounds were extracted using the UASE method. Briefly, plant powder was dispersed in ethanol solution at a preset solid-to-liquid ratio and subjected to ultrasonic extraction in an ultrasonic cleaner (Model SB-5200DTD, Ningbo Scientz Biotechnology Co., Ltd., Ningbo, China) at a fixed frequency of 40 kHz. The device was operated in intermittent mode with an actual working power of 120 W. The extraction temperature, ethanol concentration, and extraction time were varied according to the experimental designs described below. After extraction, the mixture was centrifuged (5000 rpm, 10 min), the supernatant was collected, and it was concentrated using a rotary evaporator to remove the ethanol. The resulting extract, obtained under the optimized conditions determined by RSM, was reconstituted in ultrapure water to a final volume of 6.05 mL to prepare a high-concentration stock solution rich in total phenolics (10 mg GAE/mL), which was stored at 4 °C for further use.

### 2.4. LC-MS Analysis

The total phenolic extract was analyzed using a Waters ACQUITY UPLC I-Class system coupled to a Waters XEVO-TQSmicro triple quadrupole mass spectrometer equipped with an electrospray ionization (ESI) source. Separation was performed on a Waters ACQUITY UPLC BEH C_18_ column (2.1 mm × 100 mm, 1.7 μm) maintained at 40 °C. The mobile phase consisted of 0.1% formic acid in water (A) and 0.1% formic acid in acetonitrile (B) at a flow rate of 0.4 mL/min. The gradient elution program was as follows: 0–15 min, 95–70% A; 15–19.5 min, 70–50% A; 19.5–20.5 min, 50–5% A; 20.5–21.5 min, 5% A; 21.5–22 min, 5–95% A; 22–25 min, 95% A. The injection volume was 1 μL. The mass spectrometer was operated in both positive and negative ESI modes. The capillary voltage was set at 3.0 kV, the source temperature at 150 °C, and the desolvation temperature at 500 °C. Full-scan and MS/MS spectra were acquired over a mass range of *m*/*z* 50–1000. The PDA detector was set at 254 nm.

### 2.5. Determination of the Total Phenolic Concentration

The total phenolic concentration of the extract was determined using the Folin-Ciocalteu method [26,27]. Aliquots of the gallic acid standard solution (0.1 mg/mL), ranging from 0.1 to 0.6 mL in 0.1 mL increments, were transferred to centrifuge tubes and made up to 5 mL with ultrapure water. Then, 1 mL of Folin-Ciocalteu reagent was added to each tube, and the mixture was vortexed and allowed to react for 4 min. Subsequently, 2 mL of sodium carbonate solution (75 mg/mL) was added, and the mixture was incubated at 50 °C in the dark for 5 min. After cooling to room temperature, the absorbance was measured at 765 nm, and a standard curve was constructed. The high-concentration stock solution was appropriately diluted with ultrapure water to fall within the range of the standard curve and measured using the same procedure. The total phenolic concentration of the diluted sample was calculated using gallic acid equivalents (GAE) based on the regression equation of the standard curve, and the total phenolic concentration of the stock solution (*ρ*, mg GAE/mL) was obtained by multiplying by the dilution factor. The total phenolic extraction yield was calculated as:

X (mg GAE/g) = (*ρ* × *V*/m) (1)

where X represents the total phenolic extraction yield (mg GAE/g); *ρ* is the concentration of the high-concentration stock solution of extract (mg GAE/mL); *V* is the volume of the high-concentration stock solution of extract (mL); m is the mass of the plant powder (g). These values represent GAE-based total phenolic concentrations in the crude extract and should not be interpreted as the dry mass of the crude extract or as the content of individual phenolic compounds. The concentrations used in the antioxidant and antibacterial assays were prepared by diluting this stock solution as described in the corresponding assay sections and are expressed as μg GAE/mL.

### 2.6. RSM Experimental Design

We accurately weighed 4.0 g of *T. baicalense* powder, added an ethanol solution of the corresponding concentration at a solid-to-liquid ratio of 1:10 (g:mL), and set the ultrasonic power to 120 W. With ultrasonic temperature at 40 °C, ultrasonic time at 30 min, and ethanol concentration at 50% as the baseline conditions, a single-factor experimental design was used. Two factors were fixed at the baseline levels, and the effects of ultrasonic temperature (30, 35, 40, 45, and 50 °C), ultrasonic time (30, 50, 70, 90, and 110 min), and ethanol concentration (30%, 40%, 50%, 60%, and 70%) on the total phenolic extraction yield were examined separately. After extraction, the supernatant was collected by centrifugation, and the total phenolic extraction yield was determined and calculated.

Based on the results of single-factor experiments, three independent variables-extraction temperature (30–40 °C, A), extraction time (50–90 min, B), and ethanol concentration (40–60%, C)-were selected. A three-factor, three-level Box-Behnken design (BBD) was employed to optimize the extraction process, with the total phenolic extraction yield as the response variable (Table 1 and Table 2). Design-Expert software (version 13.0) was used for regression analysis of the experimental data. A second-order quadratic polynomial regression model was established to correlate the total phenolic extraction yield with the extraction parameters.

### 2.7. Antioxidant Assays

#### 2.7.1. DPPH Radical Scavenging Activity

The DPPH radical scavenging activity was determined according to a previously described method with slight modifications [28]. Briefly, DPPH was dissolved in ethanol to prepare a 0.2 mmol/L working solution. The above high-concentration total phenolic stock solution was diluted with ethanol to 100 μg GAE/mL (final concentration 33.33 μg GAE/mL) and set aside for use. Then, 100 μL of total phenolic dilutions at different concentrations were mixed with 200 μL of the DPPH working solution. After incubation at room temperature in the dark, the absorbance was measured at 519 nm. Vitamin C was used as a positive control. The scavenging rate was calculated using the following formula:

DPPH scavenging rate (%) = (A_0_ − A_1_)/A_0_ × 100% (2)

where A_0_ is the absorbance of the blank control and A_1_ is the absorbance of the sample group.

#### 2.7.2. ABTS^+^ Radical Scavenging Activity

The ABTS^+^ radical scavenging activity was assessed following a reported method [29]. Equal volumes of 7 mmol/L ABTS solution and 2.56 mmol/L potassium persulfate (K_2_S_2_O_8_) solution were mixed and incubated in the dark at room temperature for 12 h. The resulting stock solution was then diluted with phosphate-buffered saline (PBS, pH 7.4) to an absorbance of 0.70 ± 0.02 at 734 nm. The above high-concentration total phenolic stock solution was diluted with ethanol to 100 μg GAE/mL (final concentration 20 μg GAE/mL) and set aside for use. Subsequently, 100 μL of total phenolic dilutions at different concentrations were mixed with 400 μL of the ABTS working solution, and the absorbance was recorded at 734 nm. Vitamin C served as the positive control. The ABTS^+^ scavenging rate was calculated as:

ABTS^+^ scavenging rate (%) = (A_0_ − A_1_)/A_0_ × 100% (3)

where A_0_ is the absorbance of the blank control and A_1_ is the absorbance of the sample group.

#### 2.7.3. Hydroxyl Radical (·OH) Scavenging Activity

The ·OH scavenging activity was determined using a salicylic acid system [30]. Ethanolic solutions of salicylic acid (9 mmol/L) and aqueous solutions of ferrous sulfate (9 mmol/L) were prepared. The above high-concentration total phenolic stock solution was diluted with ethanol to 100 μg GAE/mL (final concentration 3.85 μg GAE/mL) and set aside for use. Then, 20 μL of total phenolic dilutions at different concentrations were sequentially mixed with 100 μL of ferrous sulfate solution (9 mmol/L) and 100 μL of H_2_O_2_ solution (9 mmol/L). After standing at room temperature for 10 min, 300 μL of salicylic acid-ethanol solution (9 mmol/L) was added. The mixture was then incubated in a water bath at 37 °C for 30 min, and the absorbance was measured at 510 nm. Vitamin C was used as the positive control. The ·OH scavenging rate was calculated using the following formula:

(4) $$\mathrm{OH}\mathrm{scavenging}\mathrm{rate}\left(\%\right)=\left[1-\left(A_{\mathrm{sample}}-A_{\mathrm{control}}\right)/A_{\mathrm{blank}}\right]\times 100\%$$

where A_sample_ is the absorbance of the sample group, A_control_ is the absorbance of the ethanol control group, and A_blank_ is the absorbance of the blank control without sample.

### 2.8. Antibacterial Activity Assays

#### 2.8.1. Growth Inhibition Assay

The antibacterial activity of the total phenolic extracts against *E. coli* and *S. aureus* was evaluated using a growth-inhibition assay. Overnight bacterial cultures were adjusted to 1 × 10^7^ CFU/mL with LB medium. The high-concentration total phenolic stock solution was diluted with LB medium and filtered through a 0.22 μm membrane for sterilization. Then, 7 μL of bacterial suspension and 133 μL of the total phenolic solution were added to each well of a 96-well plate, giving a final inoculum of 5 × 10^5^ CFU/mL and total phenolic concentrations of 17.81, 35.63, 71.25, 142.5, 285, and 570 μg GAE/mL in a total volume of 140 μL. Untreated growth control wells contained bacterial suspension and LB medium, with the same total volume but without extract. Sterility blank wells contained only LB medium. Extract-only background wells contained LB medium and extract, without bacteria, to account for the color and turbidity of the extract. Carbenicillin (Car) was used as a positive control at concentrations numerically matching the total phenolic concentrations of the treatment groups (17.81–570 μg/mL). After incubation at 37 °C for 24 h, the optical density at 600 nm (OD_600_) of each well was measured. The bacterial inhibition rate was calculated as follows:

Inhibition rate (%) = [1 − (OD_sample_ − OD_background_)/(OD_growth control_ − OD_sterility blank_)] × 100% (5)

#### 2.8.2. Growth Kinetics Assay

To further investigate the dynamic effects of the total phenolic extract on bacterial growth, samples were prepared as described in Section 2.8.1 and incubated at 37 °C with shaking. The OD_600_ was measured every hour over a continuous 12 h period. Growth curves for *E. coli* and *S. aureus* were plotted with time (h) on the x-axis and OD_600_ on the y-axis. Untreated growth control, sterility blank, extracts-background wells, and positive control were included as described above.

#### 2.8.3. Scanning Electron Microscopy (SEM) Observation

Overnight cultures of *E. coli* and *S. aureus* were adjusted to 1 × 10^6^ CFU/mL and mixed with an equal volume of the total phenolic solution at 600 μg GAE/mL (in LB medium), resulting in a final total phenolic concentration of 300 μg GAE/mL. The mixtures were incubated at 37 °C with shaking at 220 r/min for 6 h. Cultures treated with an equal volume of LB medium instead of the extract served as the control. After incubation, the bacterial cells were harvested by centrifugation (6000 r/min, 1 min), washed three times with sterile PBS, and fixed with 2.5% glutaraldehyde at 4 °C overnight. The fixed bacterial cells were washed three times with PBS and then dehydrated through a graded ethanol series (30%, 50%, 70%, 80%, 90%, and 100%), with the 100% ethanol step repeated twice. After drying, the samples were sputter-coated with gold and observed under a scanning electron microscope.

#### 2.8.4. Crystal Violet Staining for Biofilm Quantification

Biofilm formation was assessed using a microtiter plate assay. Overnight bacterial cultures were adjusted to OD_600_ = 0.5. Aliquots (180 μL/well) of the diluted total phenol solutions (in LB medium) were added to a 96-well plate, followed by 20 μL of bacterial suspension (final volume, 200 μL), giving final total phenol concentrations of 16.9, 33.8, 67.5, 135, 270, and 540 μg GAE/mL. Negative control wells received an equal volume of LB medium instead. The plate was incubated statically at 37 °C for 48 h. The supernatant was carefully discarded, and the wells were gently washed three times with sterile PBS and air-dried at 37 °C for 6 h. Next, 150 μL of 0.1% crystal violet solution was added to each well and stained for 30 min. After rinsing with deionized water, 150 μL of 95% ethanol was added to dissolve the bound dye, followed by shaking for 30 min. Absorbance was measured at 570 nm. Based on the average OD value of the negative control (ODc), biofilm formation was classified into four categories: non-adherent (OD ≤ ODc), weakly adherent (ODc < OD ≤ 2 × ODc), moderately adherent (2 × ODc < OD ≤ 4 × ODc), and strongly adherent (OD > 4 × ODc) [31].

#### 2.8.5. Determination of Bacterial Intracellular ATP Content

Overnight cultures of *E. coli* and *S. aureus* (1 × 10^6^ CFU/mL) were mixed with an equal volume of 600 μg GAE/mL total phenolic solution, giving a final concentration of 300 μg GAE/mL. The group without the total phenolic solution served as the PBS control. And then, the mixtures were incubated at 37 °C with shaking at 220 r/min for 1 h and 3 h. After incubation, bacterial cells were harvested by centrifugation at 4 °C and 8000 r/min for 5 min, and the pellets were washed three times with sterile ice-cold PBS. The pellets were then resuspended in 50 μL PBS, and 100 μL of lysozyme (50 mg/mL) was added, followed by incubation at 37 °C for 30 min. Subsequently, 400 μL of ATP lysis buffer was added, and the mixture was incubated for 5 min. After centrifugation at 4 °C and 12,000× *g* for 20 min, the supernatant was collected, and intracellular ATP content was measured using an ATP assay kit according to the manufacturer’s instructions. The intracellular ATP content of bacteria treated with the total phenolic extract was determined to evaluate the effect of the extract on bacterial ATP levels.

### 2.9. Statistical Analysis

All experimental results were expressed as the mean ± standard deviation (SD). Three independent biological replicates were performed for each experimental group. The Box–Behnken experimental design and subsequent regression analysis were carried out using Design Expert software (version 13.0). All statistical analyses were performed using GraphPad Prism (version 9.0). Differences were considered statistically significant at *p* < 0.05.

## 3. Results and Discussion

### 3.1. Chemical Fingerprint of the Extract by LC-MS

To characterize the chemical composition of the extract, LC-MS analysis was performed. As shown in Supplementary Figure S1, the total ion chromatogram (TIC) and PDA chromatogram (254 nm) revealed multiple peaks, indicating that the extract is a complex mixture. The major PDA peaks were observed at retention times of 0.44, 2.87, 3.25, and 3.98 min, with relative peak areas of 45.61%, 18.53%, 27.98%, and 1.93%, respectively (Supplementary Table S1). The peak at 0.44 min may correspond to unretained or solvent-related compounds. The MS/MS spectra of representative peaks are shown in Supplementary Figures S2 and S3. In positive ion mode, the peak at 3.322 min exhibited a prominent fragment ion at *m*/*z* 335.96, corresponding to the major PDA peak at 3.25 min. In negative ion mode, the peak at 2.901 min showed a characteristic fragment ion at *m*/*z* 381.87, corresponding to the PDA peak at 2.87 min. These data document the chemical complexity of the extract but do not permit definitive identification of individual phenolic compounds without authentic standards or validated spectral-library matching. Given the complex chemical makeup and that the specific phenolic monomers have not been confirmed, we then measured the total phenol concentration to assess the overall phenolic level of the extract.

### 3.2. Total Phenolic Concentration Determination

**Figure 1:**
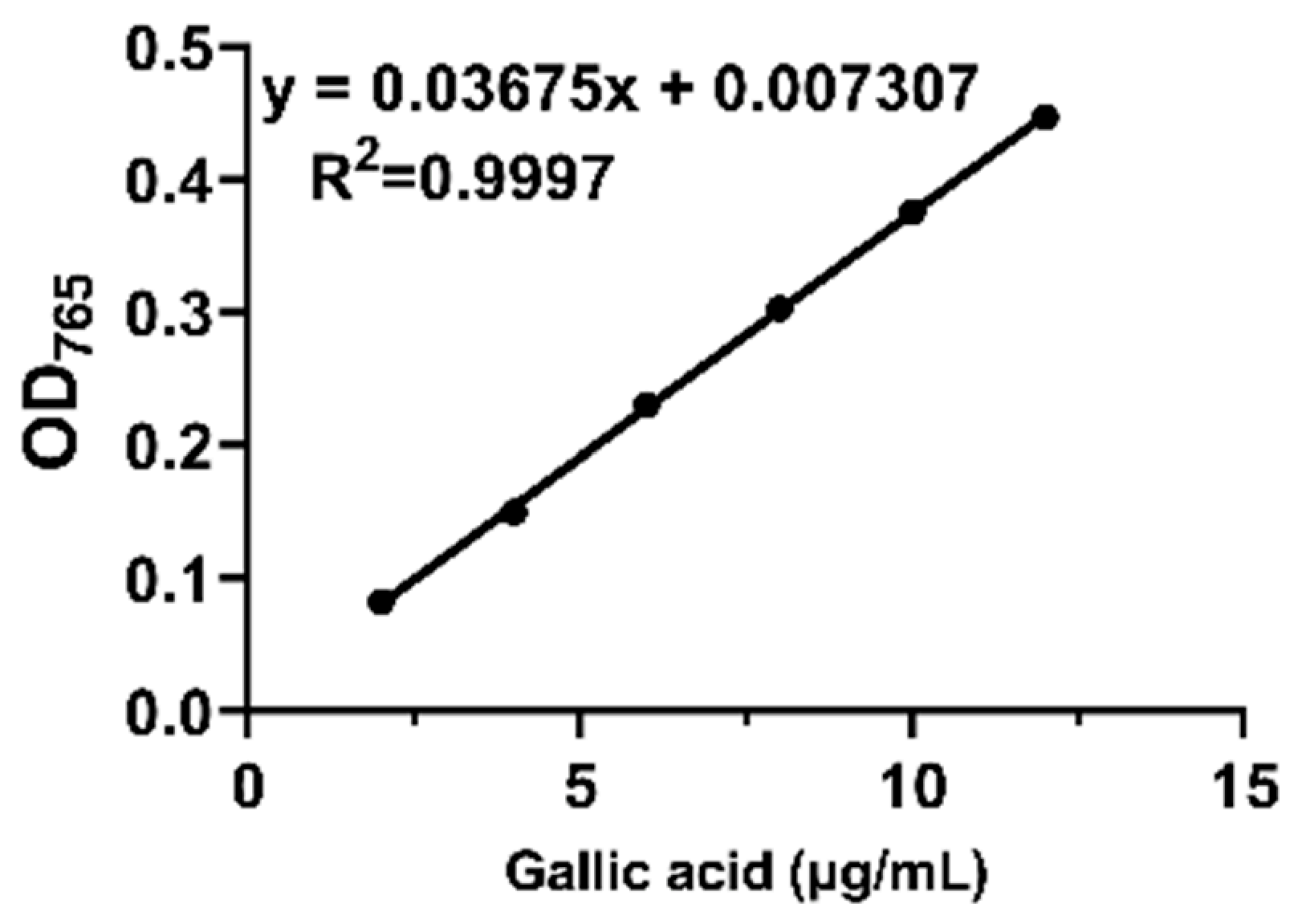
Gallic acid standard curve.

A standard curve was constructed using gallic acid as the reference standard at 765 nm (Figure 1), and the regression equation was obtained as y = 0.03675x + 0.007307 with R^2^ = 0.9997, indicating a strong linear relationship between gallic acid concentration and absorbance. The standard curve was therefore used to quantify total phenolic concentration from *T. baicalense*.

### 3.3. Single-Factor Experiments

**Figure 2:**
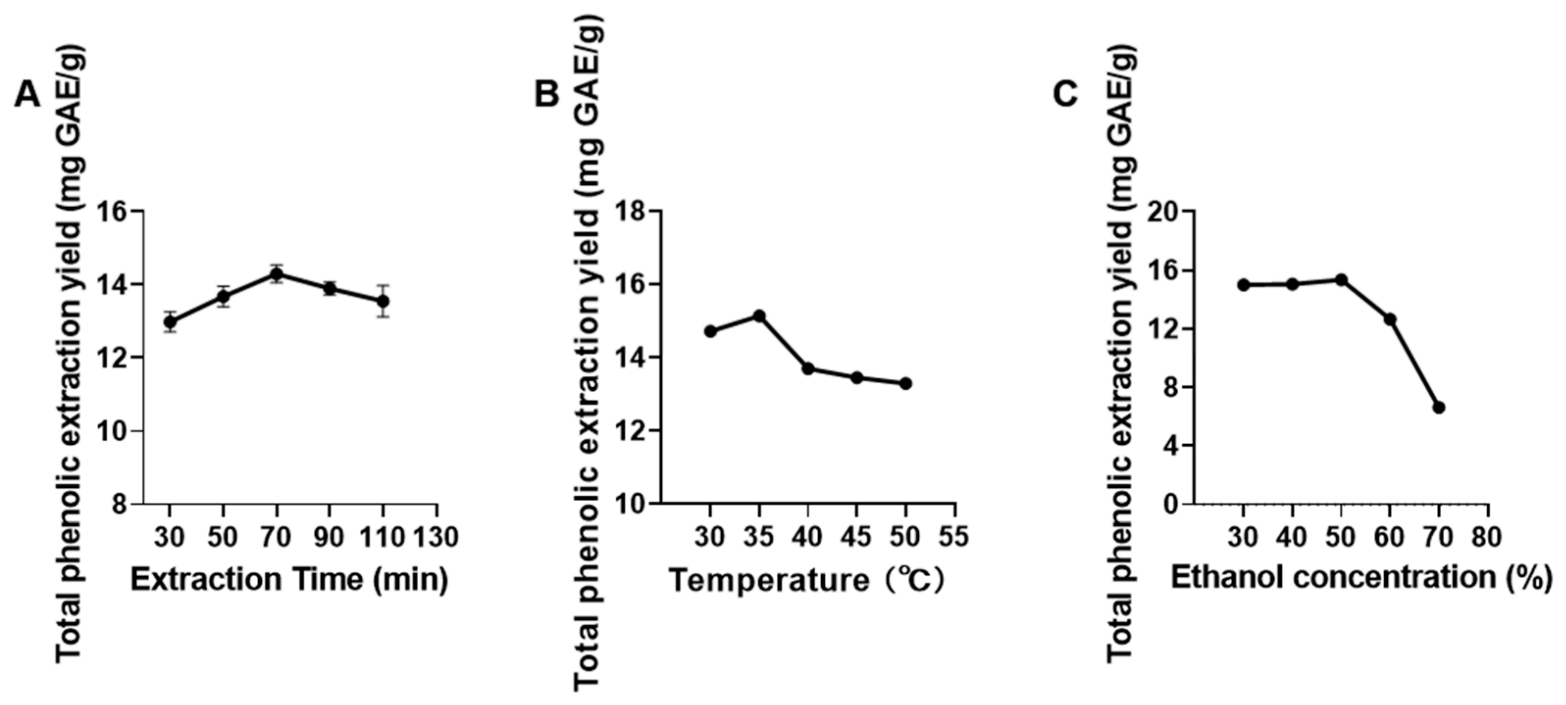
Effects of single-factor conditions on the total phenolic extraction yield from *T. baicalense.* (**A**) Extraction time; (**B**) Extraction temperature; (**C**) Ethanol concentration. Data are presented as mean ± SD (*n* = 3). Error bars represent SD.

The effects of extraction time, temperature, and ethanol concentration on the total phenolic extraction yield are presented in Figure 2A–C. As shown in Figure 2A, the total phenolic extraction yield increased when extraction time was extended from 30 to 70 min, reaching a maximum of 14.302 mg GAE/g at 70 min. The decline at longer extraction times may reflect degradation or oxidation during prolonged ultrasonication [32]. Therefore, extraction times of 50, 70, and 90 min were selected for subsequent optimization. Extraction temperature significantly influenced total phenolic extraction yield (Figure 2B), with the maximum yield observed at 35 °C. Moderate temperature elevation likely enhanced the contact frequency between solvent and phenolic molecules, thereby promoting phenolic dissolution [32,33]. However, excessive temperature might intensify solvent vaporization and cause thermal degradation of phenolics, leading to a decrease in yield. Accordingly, temperatures of 30, 35, and 40 °C were chosen for further optimization. The effect of ethanol concentration on the total phenolic extraction yield is shown in Figure 2C. In the single-factor experiment, the yield was highest at 50% ethanol and then declined at higher ethanol concentrations. This observation may be attributed to the favorable polarity match between the hydroalcoholic solvent and phenolic compounds at this concentration, which enhances extraction efficiency [34]. However, when the ethanol concentration was excessively high, the solvent polarity decreased, limiting the solubility of phenolics and thereby reducing the yield. Thus, ethanol concentrations of 40%, 50%, and 60% were selected for response surface optimization.

### 3.4. Response Surface Optimization

Based on the single-factor results, a Box-Behnken design was employed with three independent variables: extraction temperature (°C, A), extraction time (min, B), and ethanol concentration (%, C). The total phenolic extraction yield was used as the response variable. Multiple regression analysis using Design-Expert 13 software (Stat-Ease, Inc., Minneapolis, MN, USA) yielded the following second-order polynomial equation:

Y = −62.75937 + 1.84250A + 0.416603B + 1.36615C − 0.002542AB − 0.006617AC − 0.000488BC − 0.021092A^2^ − 0.002111B^2^ − 0.011507C^2^ (6)

The ANOVA and regression coefficient test results for the response surface model are given in Table 3 and Table 4. The model F-value of 59.15 with *p* < 0.0001 indicated that the model was highly significant. The lack-of-fit test yielded *p* > 0.05, confirming that the model adequately fitted the experimental data without significant lack of fit. The F-values revealed that the factors affected the total phenolic extraction yield in the following order: extraction temperature (A) > ethanol concentration (C) > extraction time (B). The coefficients A, B^2^, and C^2^ exhibited highly significant effects on the total phenolic extraction yield, while AC and A^2^ also showed significant effects. The interaction term AB had a relatively smaller but still significant effect. The predicted R^2^ (R^2^ = 0.8295) and adjusted R^2^ (R^2^_adj_ = 0.9703) differed by less than 0.2, indicating good consistency between predicted and actual values, thereby confirming the reliability of the model predictions.

### 3.5. Response Surface Analysis

**Figure 3:**
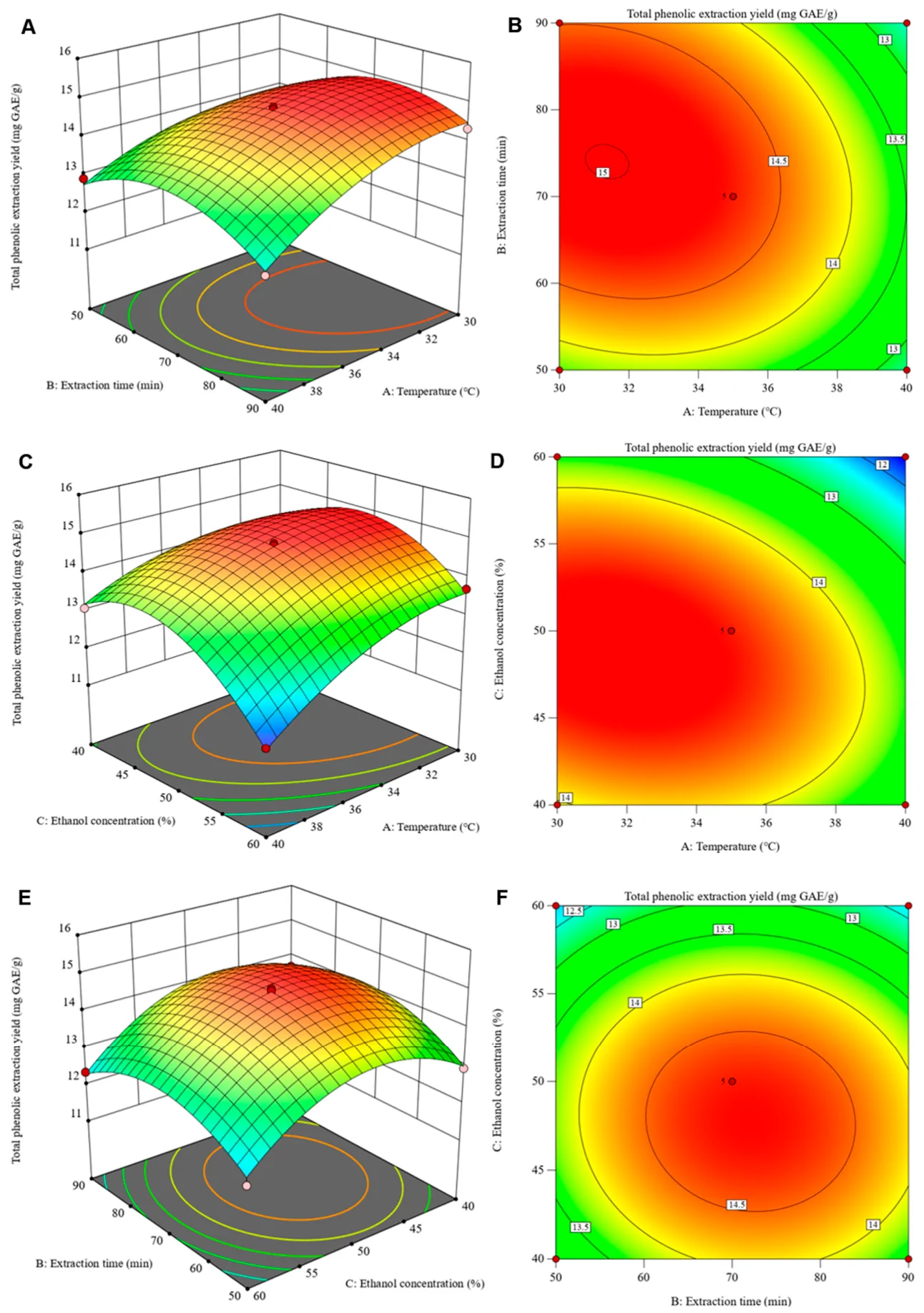
Response surface and contour plots for the effects of temperature, time, and ethanol concentration on the total phenolic extraction yield from *T. baicalense.* (**A**,**B**) Temperature and time; (**C**,**D**) Temperature and ethanol concentration; (**E**,**F**) Time and ethanol concentration.

The three-dimensional response surface plots and corresponding contour plots (Figure 3A–F) visually illustrated the interactive effects of the three variables on the total phenolic extraction yield. Figure 3A,B showed that at a fixed ethanol concentration, increasing the extraction temperature from 30 to 32.29 °C and extending the extraction time from 50 to 72.11 min resulted in an increased total phenolic extraction yield. Figure 3C,D revealed that at a fixed extraction time, raising the ethanol concentration from 40 to 48.61% and temperature from 30 to 32.29 °C improved the yield. Figure 3E,F demonstrated that at a fixed extraction temperature, the total phenolic extraction yield reached a maximum when the ethanol concentration ranged from 40 to 48.61% and the extraction time ranged from 50 to 72.11 min.

### 3.6. Model Validation

The model predicted optimal extraction conditions of 72.113 min, 32.290 °C, and 48.614% ethanol, with a predicted total phenolic extraction yield of 15.005 mg GAE/g. For practical convenience, these conditions were adjusted to 72 min, 32 °C, and 49% ethanol. Three independent verification experiments under the adjusted conditions yielded a mean total phenolic extraction yield of 15.116 mg GAE/g (RSD = 0.13%), which was 0.111 mg/g (0.74%) higher than the predicted value. Based on the residual standard deviation (0.1828), residual degrees of freedom (7), and the leverage of the prediction point (0.194), the 95% prediction interval was calculated to be 14.53–15.48 mg GAE/g. The observed mean fell within this interval, confirming the reliability of the model within the studied design space.

The verification yield was slightly lower (by 0.460 mg GAE/g, approximately 2.95%) than the highest yield obtained in the single-factor ethanol experiment (15.576 mg GAE/g). The single-factor maximum was obtained at 40 °C and 30 min, whereas the Box-Behnken design covered a time range of 50–90 min and did not include the 30 min condition. Therefore, the RSM optimum represents the best combination within the selected design space rather than a global maximum across all possible conditions. These results indicate that the model is reliable for predicting total phenolic extraction within the studied ranges, although further optimization may be warranted if broader conditions are considered.

### 3.7. Antioxidant Activity

**Figure 4:**
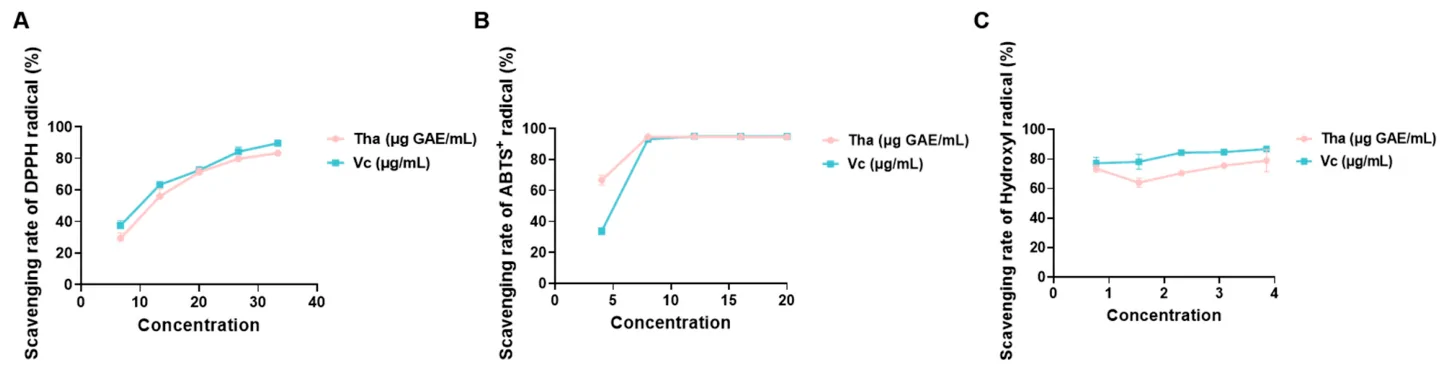
Antioxidant activity of total phenolic extract from *T. baicalense.* (**A**) Scavenging effect on DPPH radicals; (**B**) Scavenging effect on ABTS^+^ radicals; (**C**) Scavenging effect on ·OH radicals. (Tha: the total phenolic extract from *T. baicalense*; Vc: Vitamin C.) Data are presented as mean ± SD (*n* = 3). Error bars represent SD.

The antioxidant activities of the total phenolic extract from *T. baicalense* are presented in Figure 4. As shown in Figure 4A, the DPPH radical scavenging activity of the total phenolic extract increased with increasing concentration in the range of 6.67–33.33 μg GAE/mL, reaching a maximum of (83.41 ± 0.37)% at 33.33 μg GAE/mL. This value was slightly lower than that of Vc (89.66 ± 0.38%) at the same concentration, but superior to previously reported values for related species [9]. The ABTS^+^ radical scavenging activity (Figure 4B) increased with concentration up to 8 μg GAE/mL, reaching a peak of 94.70%, slightly higher than that of Vc (93.10%). At concentrations ranging from 12 to 20 μg GAE/mL, the scavenging activity remained stable between 94.51% and 94.69%, comparable to Vc (94.97–95.01%). The ·OH scavenging activity (Figure 4C) ranged from 73.43% to 78.95% at concentrations of 0.77–3.85 μg GAE/mL, which was similar to that of Vc (77.19–86.76%), indicating moderate ·OH scavenging capacity.

### 3.8. Antimicrobial Activity

**Figure 5:**
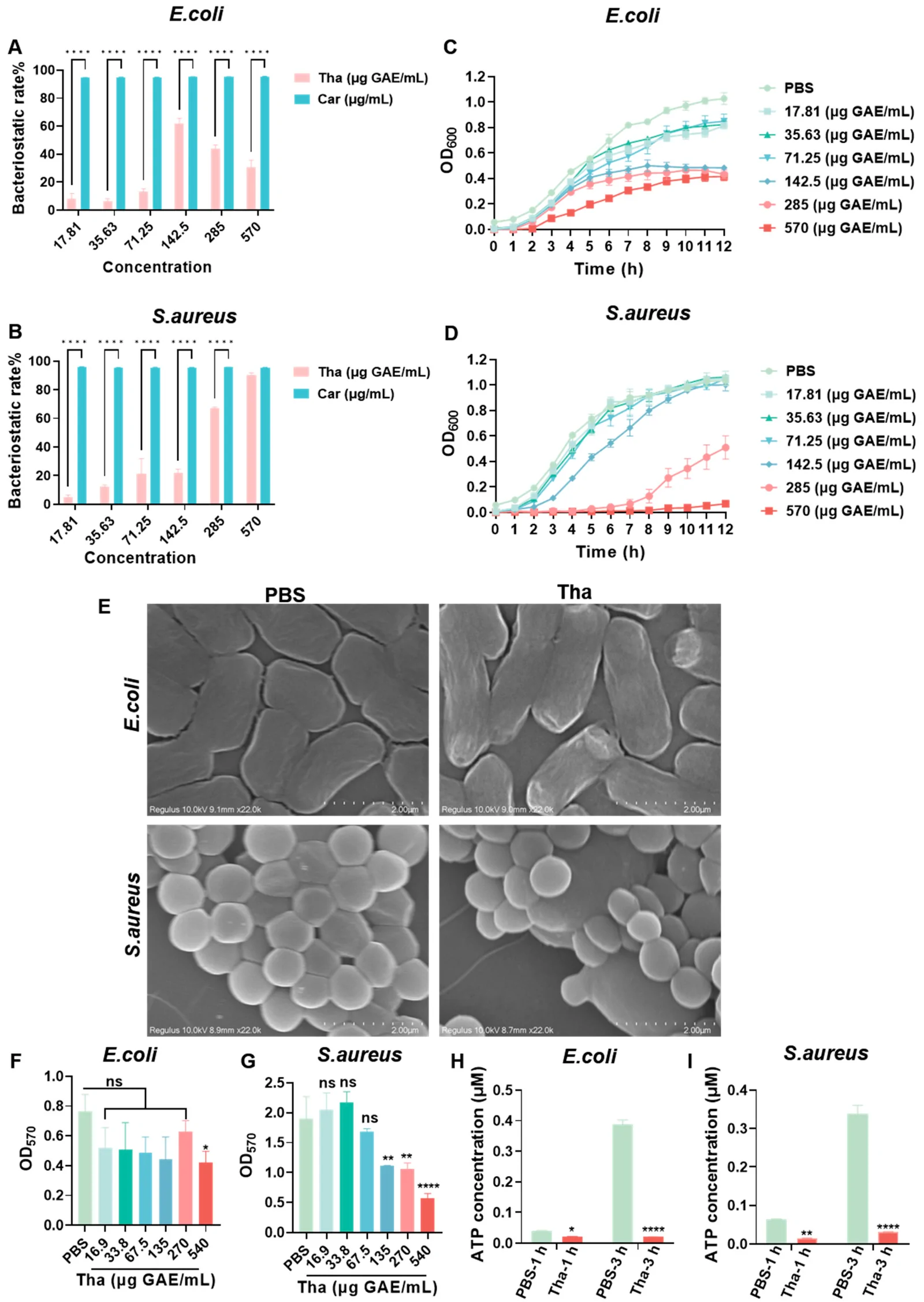
Antibacterial activity of the total phenolic extract from *T. baicalense.* (**A**,**B**) Inhibition rates against *E. coli* and *S. aureus*; (**C**,**D**) Effects on the growth of the two strains; (**E**) Effects on the morphology of the two strains (SEM); (**F**,**G**) Inhibition of biofilm formation by the two strains; (**H**,**I**). Reduction of intracellular ATP levels by the total phenolic extract from *T. baicalense* in the two strains. Data are presented as mean ± SD (*n* = 3). Error bars represent SD. Statistical significance for A, B, H, and I was determined by two-way ANOVA followed by Tukey’s multiple-comparison test and for F and G by one-way ANOVA followed by Dunnett’s multiple-comparison test. * *p* < 0.05, ** *p* < 0.01, **** *p* < 0.0001; ns, not significant.

The antimicrobial activity of the total phenolic extract against *E. coli* and *S. aureus* is shown in Figure 5. The inhibition rate against *E. coli* increased when increasing the total phenolic concentration up to 142.5 μg GAE/mL, beyond which no significant differences were observed among concentrations of 142.5, 285, and 570 μg GAE/mL (*p* > 0.05) (Figure 5A). Growth curve analysis (Figure 5C) confirmed that *E. coli* growth was markedly inhibited at concentrations ≥ 142.5 μg GAE/mL after 10–12 h of incubation. For *S. aureus*, the total phenolic extracts exhibited more pronounced growth inhibitory activity (Figure 5B), achieving an inhibition rate of (90.34 ± 0.1)% at 570 μg GAE/mL, which was comparable to that of the positive control Car at the same concentration (*p* > 0.05). Growth curve analysis (Figure 5D) showed that the OD_600_ value approached 0.1 at 570 μg GAE/mL, indicating notable growth inhibition, consistent with the inhibition rate results. These findings were in agreement with previously reported antimicrobial activities of related *Thalictrum* species extracts [9].

SEM revealed treatment-associated morphological changes (Figure 5E). Untreated *E. coli* cells displayed typical rod-shaped morphology and smooth cell surfaces, whereas cells treated with the total phenolic extract at a final concentration of 300 μg GAE/mL for 6 h exhibited obvious morphological damage and structural disintegration. Similarly, untreated *S. aureus* cells showed a typical spherical morphology and smooth surfaces, whereas treated cells displayed aggregation and swelling. These observations are consistent with treatment-associated cell surface damage but do not, by themselves, establish increased membrane permeability.

The biofilm formation inhibitory activity was quantitatively assessed using crystal violet staining (Figure 5F,G). The total phenolic extract significantly inhibited *E. coli* biofilm formation at 540 μg GAE/mL (*p* < 0.05). For *S. aureus*, biofilm biomass was significantly reduced at 135 and 270 μg GAE/mL (*p* < 0.01), and highly significant inhibition was observed at 540 μg GAE/mL (*p* < 0.0001). Collectively, these results indicate that the total phenolic extract from *T. baicalense* reduced biofilm biomass under the tested conditions. Because the tested concentrations also inhibited planktonic growth, the present data cannot distinguish a specific antibiofilm effect from general growth inhibition. Further studies using sub-inhibitory concentrations are needed to confirm whether the total phenolic extract from *T. baicalense* has a specific antibiofilm effect.

Intracellular ATP was measured as an indicator of bacterial cellular energy status [35] (Figure 5H,I). After treatment with the total phenolic extract at a final concentration of 300 μg GAE/mL for 1 h and 3 h, the intracellular ATP concentrations in both *E. coli* and *S. aureus* were significantly reduced compared with those in the PBS control group. For *E. coli*, the ATP concentrations in the PBS control group at 1 h and 3 h were approximately 0.04 μM and 0.39 μM, respectively, whereas those in the total phenolic extract-treated groups at the corresponding time points were all below 0.03 μM. For *S. aureus*, the ATP concentrations in the PBS control group at 1 h and 3 h were approximately 0.07 μM and 0.34 μM, respectively, whereas those in the treated groups at 1 h and 3 h were all reduced to approximately 0.02 μM, with all reductions reaching statistical significance. These results indicate that the total phenolic extract from *T. baicalense* can reduce intracellular ATP in *E. coli* and *S. aureus*. However, whether this reduction reflects a direct effect on energy metabolism or is a consequence of growth inhibition and cell damage requires further investigation.

## 4. Conclusions

In this study, the LC-MS profile showed that the extract was chemically complex, but definitive identification of individual phenolic compounds requires authentic standards or validated spectral matching. This compositional limitation should be considered alongside the Folin-Ciocalteu results. The total phenolic extraction yield from *T. baicalense* was optimized by UASE in conjunction with RSM. Single-factor experiments were conducted to preliminarily determine the extraction conditions, followed by response surface design to optimize the process parameters. The optimal extraction conditions were identified as: extraction time of 72 min, extraction temperature of 32 °C, and ethanol concentration of 49% (*v*/*v*). Under these conditions, the total phenolic extraction yield reached 15.116 mg GAE/g, which was in good agreement with the model-predicted value, confirming the reliability and effectiveness of the extraction process. The antioxidant activity evaluation demonstrated that the total phenolic extract exhibited strong scavenging activity against DPPH, ABTS^+^, and ·OH, with efficacy comparable to that of Vc at the same concentration. Antimicrobial activity assays revealed that the total phenolic extract inhibited *S. aureus*, with an inhibition rate of (90.34 ± 0.1)% at a concentration of 570 μg GAE/mL. Although this inhibition was not significantly different from that of the positive control Car, this does not establish comparable efficacy. The extract exhibited weaker activity against *E. coli*. Mechanistic studies suggested that the antibacterial effects of the total phenolic extract may involve reduced intracellular ATP levels and decreased biofilm biomass, indicating potential antibiofilm activity. However, these results do not establish a specific antibiofilm mechanism, and the specific mechanisms and actual efficacy require further confirmation. In summary, the total phenolic extract from *T. baicalense* exhibited promising antioxidant activity and notable anti-*S. aureus* activity against the tested strain (s) in vitro. These findings suggest that the total phenolic extract from *T. baicalense* has potential as a natural antioxidant and antimicrobial agent. Although practical applications in food and pharmaceuticals require further validation through in vivo studies, broader-spectrum testing, and safety evaluation, the present study provides a preliminary experimental basis for their further development.

## Figures and Tables

**Table 1:** Range of variables at different levels for response surface optimization.

Independent Variable	Symbol	Coded Levels		
		−1	0	1
Extraction temperature (°C)	A	30	35	40
Extraction time (min)	B	50	70	90
Ethanol concentration (%)	C	40	50	60

**Table 2:** Response surface experimental design.

Trial no.	Levels		
	A	B	C
1	−1	−1	0
2	1	−1	0
3	−1	1	0
4	1	1	0
5	−1	0	−1
6	1	0	−1
7	−1	0	1
8	1	0	1
9	0	−1	−1
10	0	1	−1
11	0	−1	1
12	0	1	1
13	0	0	0
14	0	0	0
15	0	0	0
16	0	0	0
17	0	0	0

**Table 3:** Response surface experimental design and data.

Run	Levels			Total Phenolic Extraction Yield/(mg GAE/g)	
	A	B	C	Actual	Prediction
1	−1	−1	0	13.77	13.67
2	1	−1	0	12.91	12.75
3	−1	1	0	14.33	14.49
4	1	1	0	12.45	12.55
5	−1	0	−1	13.89	13.94
6	1	0	−1	13.07	13.18
7	−1	0	1	13.70	13.60
8	1	0	1	11.55	11.51
9	0	−1	−1	12.93	12.99
10	0	1	−1	13.70	13.60
11	0	−1	1	11.97	12.18
12	0	1	1	12.35	12.29
13	0	0	0	14.76	14.73
14	0	0	0	14.55	14.73
15	0	0	0	14.79	14.73
16	0	0	0	14.74	14.73
17	0	0	0	14.83	14.73

**Table 4:** Analysis of variance for response surface model.

Source of Variation	Sum of Squares	Degrees of Freedom	Mean Square	F Value	*p* Value	Significance
Model	17.79	9	1.98	59.15	<0.0001	***
A	4.08	1	4.08	122.06	<0.0001	***
B	0.1922	1	0.1922	5.75	0.0476	*
C	2.03	1	2.03	60.65	0.0001	**
AB	0.2586	1	0.2586	7.74	0.0272	*
AC	0.4379	1	0.4379	13.11	0.0085	**
BC	0.0380	1	0.0380	1.14	0.3214	
A^2^	1.17	1	1.17	35.04	0.0006	**
B^2^	3.00	1	3.00	89.82	<0.0001	***
C^2^	5.58	1	5.58	166.87	<0.0001	***
Residual	0.2339	7	0.0334			
Lack of Fit	0.1875	3	0.0625	5.40	0.0686	ns
Pure Error	0.0463	4	0.0116			
Cor total	18.02	16				
SD	0.1828		R^2^	0.9870		
Mean	13.55		Adj- R^2^	0.9703		
C. V. %	1.35		Pred- R^2^	0.8295		
			Adeq Precision	23.0128		

Note: *** indicates extremely significant differences (*p* < 0.0001); ** indicates relatively significant differences (*p* < 0.01); * indicates significant differences (*p* < 0.05); ns indicates not significant.

## Data Availability

The original contributions presented in the study are included in the article/Supplementary Material. Further inquiries can be directed to the corresponding author.

## Supplementary Materials

The following supporting information can be downloaded at: https://www.mdpi.com/article/10.3390/pathogens15101088/s1, Figure S1: TIC (positive and negative ion modes) and PDA chromatogram (254 nm) of the extract; Table S1: Peak list from the PDA chromatogram (254 nm) of the extract from *T. baicalensee*; Figure S2: Positive ion mode (ESI+) MS/MS spectrum of the peak at 3.322 min in the extract of *T. baicalense*, showing a major fragment ion at *m*/*z* 335.96. This peak corresponds to the major PDA peak at 3.25 min; Figure S3: Negative ion mode (ESI−) MS/MS spectrum of the peak at 2.901 min in the extract of *T. baicalense*, showing a characteristic fragment ion at *m*/*z* 381.87. This peak corresponds to the major PDA peak at 2.87 min.
