# Supplementary material for: Optimization of Extraction Process and Antioxidant and Antibacterial Activities of Total Phenolics from Thalictrum baicalense Turcz
Source: Pathogens. 2026 Oct 16;15(10):1088. doi: 10.3390/pathogens15101088 (PMC13708015; doi:10.3390/pathogens15101088)
Supplement: Supplementary file 1 [file pathogens-15-01088-s001.zip › pathogens-4580087-supplementary.pdf]

Supporting information

Optimization of Extraction Process and Antioxidant and Antibacterial Activities of Total Phenolics from *Thalictrum baicalense* Turcz

Lili Wu[1], Boyuan Liu[1], Jiawen Yao[1], Sha Li[1,*]

[1] School of Bioengineering, Zunyi Medical University, Zhuhai, Guangdong 519041, P. R. China

* Corresponding author. e-mail addresses: lisha_jnu@163.com (S. Li)

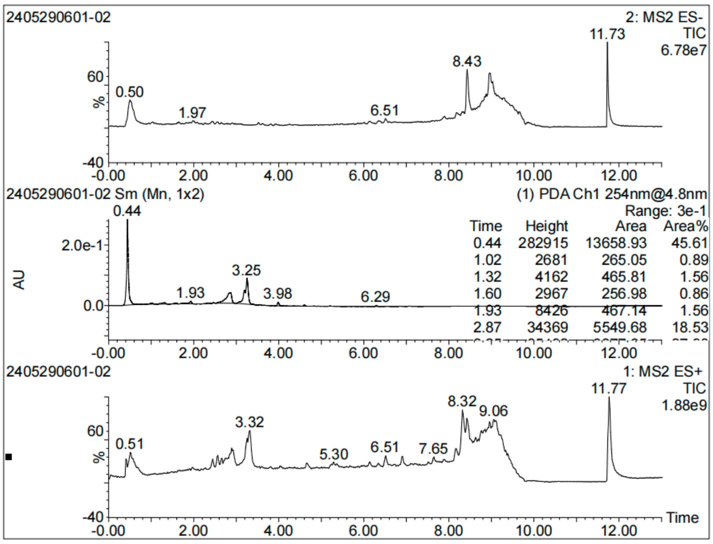

Figure S1. TIC (positive and negative ion modes) and PDA chromatogram (254 nm) of the extract.

Table S1. Peak list from the PDA chromatogram (254 nm) of the extract from *T. baicalense*.

Peak No.	Retention time (min)	Height	Area	Area (%)
1	0.44	282915	13658.93	45.61
2	1.02	2681	265.05	0.89
3	1.32	4162	465.81	1.56

Peak No.	Retention time (min)	Height	Area	Area (%)
4	1.60	2967	256.98	0.86
5	1.93	8426	467.14	1.56
6	2.87	34369	5549.68	18.53
7	3.25	85492	8377.35	27.98
8	3.98	12355	579.02	1.93
9	4.60	4534	193.09	0.64
10	6.29	3266	131.21	0.44

Note: Peak height and peak area are expressed in arbitrary units (AU). The PDA detector was set at 254 nm. The data were obtained from the same LC-MS run as shown in Figure S1.

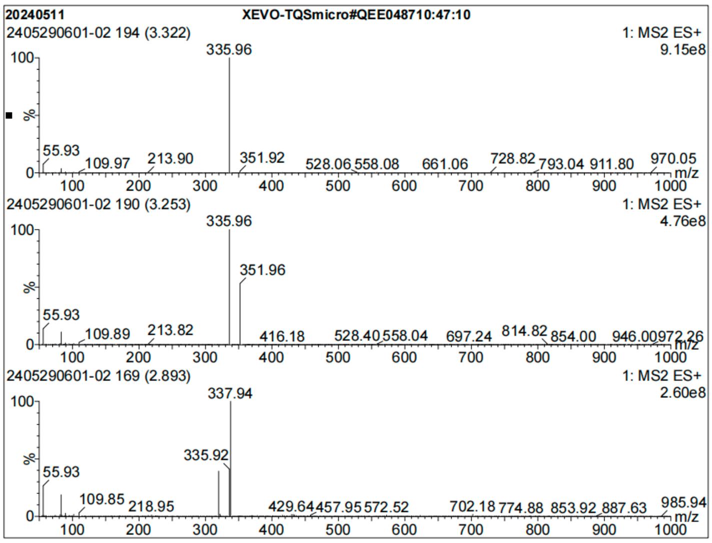

Figure S2. Positive ion mode (ESI+) MS/MS spectrum of the peak at 3.322 min in the extract of *T. baicalense*, showing a major fragment ion at *m/z* 335.96. This peak corresponds to the major PDA peak at 3.25 min.

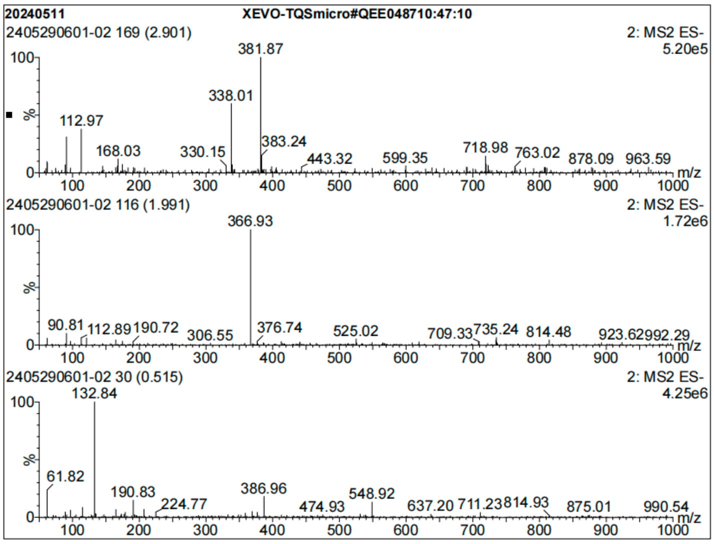

Figure S3. Negative ion mode (ESI−) MS/MS spectrum of the peak at 2.901 min in the extract of *T. baicalense*, showing a characteristic fragment ion at *m/z* 381.87. This peak corresponds to the major PDA peak at 2.87 min.
